# Posterior Circulation Stroke in a Patient With Giant Cell Arteritis: A Case Report

**DOI:** 10.7759/cureus.104110

**Published:** 2026-02-23

**Authors:** Sonakshi Nemchand, Ling Fung Chan, Siddharth Munsuri, Sameer Almashta

**Affiliations:** 1 Internal Medicine, Wrexham Maelor Hospital, Wrexham, GBR; 2 Stroke Consultancy, Wrexham Maelor Hospital, Wrexham, GBR

**Keywords:** atrial fib, corticosteroids, giant-cell arteritis, posterior circulation stroke, stroke rehab

## Abstract

We present the case of a 95-year-old woman with recently diagnosed giant cell arteritis (GCA) who developed a posterior circulation stroke. She initially experienced visual disturbances and was treated with high-dose corticosteroids after a temporal artery ultrasound confirmed the diagnosis. Two weeks later, she developed sudden left-sided weakness and unsteadiness. Imaging revealed an acute infarct in the left cerebellar hemisphere. During admission, atrial fibrillation was also detected. This case highlights the diagnostic challenge of stroke in GCA, where both vascular inflammation and coexisting cardioembolic risk factors can contribute to cerebrovascular events.

## Introduction

Giant cell arteritis (GCA) is a systemic vasculitis affecting medium- to large-sized arteries, particularly the cranial branches of the external carotid artery [1]. It predominantly affects individuals over 50 years of age and is more common in women. Typical manifestations include headache, scalp tenderness, jaw claudication, and visual disturbances. Although cerebrovascular complications are less frequent, they can be severe, particularly when involving the vertebrobasilar circulation [2,3].

A stroke occurring shortly after the initiation of high-dose corticosteroids presents a clinical dilemma about whether the mechanism is persistent inflammatory vasculopathy, cardioembolism, or a combination of both. This case describes a 95-year-old woman who developed a posterior circulation stroke shortly after a new diagnosis of GCA, in the context of newly detected atrial fibrillation.

## Case presentation

**Figure 1:**
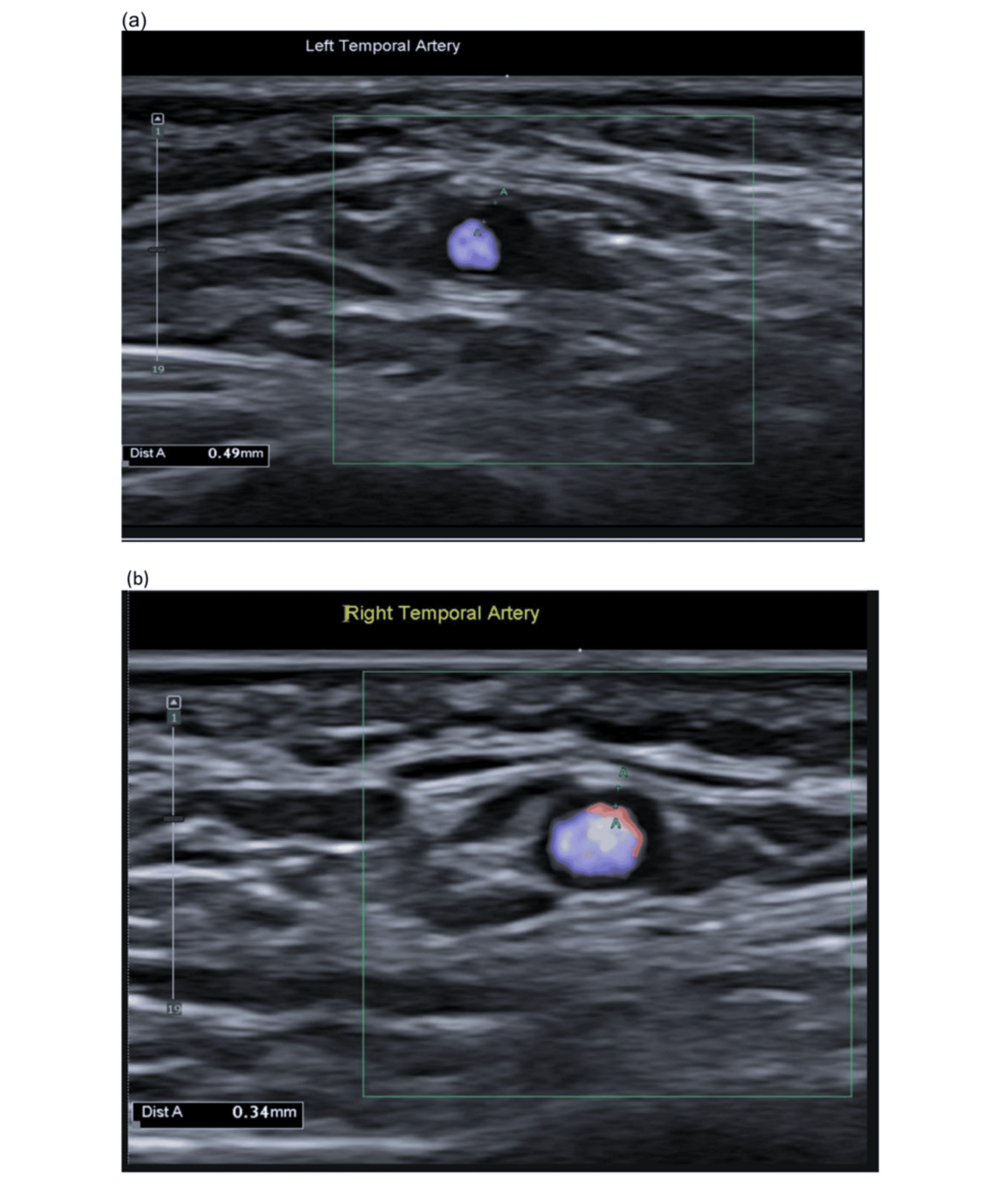
a-b: Temporal artery ultrasound showing halo signs in the left (0.49 mm) and right (0.34 mm) temporal arteries, including the involvement of the right parietal branch, consistent with inflammatory changes seen in giant cell arteritis.

A 95-year-old woman initially presented with visual hallucinations, color vision disturbances, and left-sided temporal headache, accompanied by intermittent pain below the left ear. Although C-reative protein (CRP) was only mildly raised (6 mg/L), her temporal artery ultrasound revealed halo signs in both temporal arteries - left 0.49 mm, right 0.34 mm, including involvement of the right parietal branch--consistent with GCA (Figures 1a-1b). She was started on oral prednisolone 60 mg daily and tapered down 5 mg every 5 days. Over the next few days, her visual symptoms improved, and she was discharged home.

The inflammatory markers at diagnosis were CRP: 1 mg/L (normal) and hemoglobin: 136 g/L (normal). ESR was not performed at the time of diagnosis. The patient's past medical history included hypertension, deep vein thrombosis, and previous cataract surgery.

**Figure 2:**
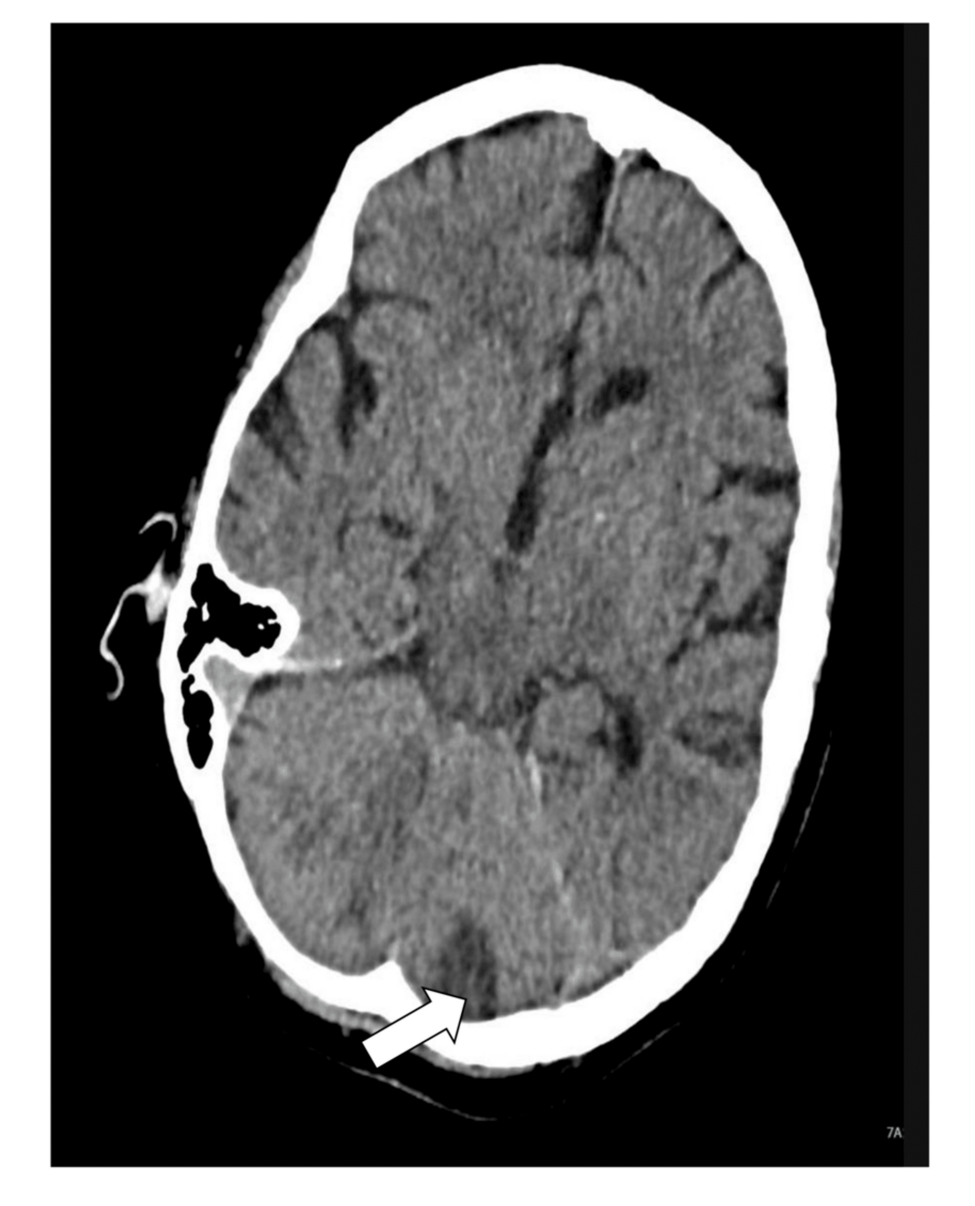
Axial CT brain demonstrating an acute infarct in the posterior aspect of the left cerebellar hemisphere, consistent with a posterior circulation stroke (white arrow)

She subsequently presented with acute neurological symptoms. CT brain demonstrated an acute infarct in the posterior circulation territory (Figure 2). She was reviewed by the stroke consultant and diagnosed with an acute posterior circulation stroke, embolic in nature, with vertebral artery obstruction identified.

Telemetry monitoring during admission revealed atrial fibrillation, which had not previously been documented in her medical records. It was therefore treated as newly detected atrial fibrillation. Her CHA₂DS₂-VASc score was calculated as 6 (age ≥75 years, hypertension, female sex, and current stroke), indicating a high thromboembolic risk. Her renal function was preserved (eGFR >90 mL/min/1.73m²), and she was commenced on apixaban for anticoagulation following a multidisciplinary discussion. She was also noted to have severe vitamin D deficiency and was started on replacement therapy.

Repeat inflammatory markers during admission remained unremarkable. Rheumatology review recommended continuation of high-dose corticosteroids with tapering. Thyroid function tests were initially deranged; endocrinology review and thyroid ultrasound demonstrated benign findings, and repeat TFTs normalized without intervention.

Hospital course and outcome

During admission, she received regular physiotherapy and occupational therapy input. Her neurological deficits remained stable without deterioration. She showed gradual improvement in mobility and functional status. She was discharged to Deeside Hospital for ongoing rehabilitation.

She remained under rheumatology follow-up, with an outpatient review scheduled for 17/03/2025. At follow-up, she was clinically stable and continuing rehabilitation with no documented recurrent cerebrovascular events.

## Discussion

Posterior circulation stroke in GCA is an uncommon but clinically significant event. While visual disturbances are hallmark features of GCA, vertebrobasilar involvement is disproportionately represented among GCA-related strokes [4,5].

The incidence of stroke in GCA varies across studies. Population-based data suggest a 2-4% overall risk, but some cohorts report higher hazard ratios for cerebrovascular accidents in GCA patients compared to controls [6,7]. Importantly, the risk appears greatest in the early phase of treatment, raising the possibility that corticosteroids, while effective for controlling vascular inflammation, do not immediately eliminate thromboembolic risk [3,8].

Atrial fibrillation (AF) complicates the picture, given its independent association with ischemic stroke. The coexistence of AF and GCA underscores the need for a comprehensive approach that balances immunosuppression, anticoagulation, and secondary prevention measures.

The National Institute for Health and Care Excellence (NICE) has issued clear guidance on the management of patients with AF who have suffered a stroke, recommending the initiation of long-term anticoagulation to reduce the risk of recurrent cerebrovascular events. In contrast, stroke associated with giant cell arteritis (GCA) is rare, and consequently, there are no established evidence-based guidelines or consensus recommendations to inform its management [2]. Prior observational studies have suggested that adjunctive antiplatelet or anticoagulant therapy may reduce ischemic complications in GCA [9,10].

Learning points

The following points can be learned from this case: 1. Posterior circulation stroke can occur early after GCA diagnosis despite the initiation of high-dose corticosteroids; 2. Normal CRP does not exclude active GCA, particularly when imaging confirms halo signs; 3. Newly detected atrial fibrillation should prompt a CHA₂DS₂-VASc assessment and anticoagulation where appropriate; 4. Stroke in GCA may be inflammatory, embolic, or multifactorial; distinguishing mechanisms can be challenging in elderly patients; 5. Clear documentation of hospital course and follow-up strengthens case reporting and outcome interpretation.

## Conclusions

This case illustrates the complex interplay between inflammatory vasculopathy and cardioembolic risk in a very elderly patient with GCA. The occurrence of posterior circulation stroke despite high-dose corticosteroid therapy emphasizes that early cerebrovascular complications remain possible.

The presence of newly detected atrial fibrillation further increased thromboembolic risk, necessitating anticoagulation. In elderly patients with overlapping risk factors, the stroke mechanism may be multifactorial, requiring individualized and multidisciplinary management. Early vigilance, comprehensive stroke workup, and coordinated follow-up are essential to optimize outcomes in this rare but clinically significant scenario.
